# Reliability and validity of the Japanese version of the weight bias internalization scale

**DOI:** 10.1186/s13104-022-06221-x

**Published:** 2022-10-22

**Authors:** Shota Endo, Hideaki Kasuga, Masuishi Yusuke, Tomoo Hidaka, Takeyasu Kakamu, Tetsuhito Fukushima

**Affiliations:** Department of Hygiene and Preventive Medicine, Fukushima Medical University School of Medicine, Hikarigaoka 1, 960-1295 Fukushima, Japan

**Keywords:** Obesity, Weight bias, Japanese

## Abstract

**Objective:**

The weight bias internalization scale (WBIS) is widely used in several languages. However, there is no Japanese version of the WBIS. The purpose of this study is to develop a Japanese version of the WBIS and to verify its reliability and validity. We translated the original version of the WBIS and had approval from the scale developer. Adults who perceived themselves to be obese completed online baseline survey (N = 285) and two-week follow-up survey (N = 100). We used the Japanese WBIS and scales for obesity-related quality of life, self-esteem, self-efficacy, and depression. We calculated Cronbach’s alpha and intra-class correlation coefficient (ICC) to assess reliability of the WBIS and conducted confirmatory factor analysis (CFA) and correlation analysis to assess its validity.

**Results:**

Cronbach’s alpha was 0.91 at baseline and 0.92 at follow-up, and the ICC was 0.87. CFA showed that a one-factor model demonstrated an acceptable fit (χ2 (44) = 158.6, CFI = 0.93, RMSEA = 0.096, SRMR = 0.051), similar to the original version. As we had hypothesized, overall, the Japanese version of the WBIS was significantly correlated with obesity-related quality of life, self-esteem, self-efficacy, and depression. These results confirmed its adequate reliability and validity.

**Supplementary Information:**

The online version contains supplementary material available at 10.1186/s13104-022-06221-x.

## Introduction

People with obesity are highly stigmatized and face discrimination and prejudice because of their weight [1]. The weight stigma about obesity is thought to be rooted in the misconception that body weight is easily controlled by changing the diet and physical activity levels [2]. Weight stigma internalization refers to “internalization of negative weight stereotypes and subsequent self-disparagement” [3]. Weight stigma internalization is associated with poor psychological [3–5], physical [5–8], and social [8–10] outcomes. Therefore, it is important for healthcare professionals [8, 11], social media [12, 13], and public health policy makers [14] not to exacerbate weight bias.

There are several self-reported questionnaires about internalized weight bias, among which weight bias internalization scale (WBIS) [15] is the most used. A previous study compared the WBIS and the Weight Self-Stigma Questionnaire and showed a higher reliability of the former [16]. The original version of the WBIS is in English, and it has been translated into several languages [17–19], including Asian languages [20]. However, a Japanese version of the WBIS has not yet been developed.

In Japan, the government began a health checkup for abdominal obesity in 2008, but the prevalence of obesity has not decreased between 2009 and 2019, with approximately 30% in males and 20% in females [21]. Although the health checkup has promoted awareness of obesity, previous studies have raised concerns that awareness-based approaches to obesity prevention could lead to unintended consequences from weight stigma [22, 23]. Japanese young females perceived high pressure from social media to be thin [24] and, dissatisfied with and concerned about their bodies [25]. In Japanese adolescents, 16% of males and 32% of females experienced body-related teasing in school and/or at home, and the experience associated with the perception to be overweight [26]. Despite this society-wide obsession against obesity, little is known about the potential harm of weight bias internalization in Japan. Weight bias toward individuals has been shown to exist in Japan as in other countries [27]. A previous study has shown that weight bias internalizes individuals when they perceived negative responses from others regarding their weight or body shape [3]. We hypothesized that weight bias internalization can play an important role in Japan. The aim of this study is to develop a Japanese version of the WBIS and to verify its reliability and validity.

## Methods

### Participants

We conducted a baseline and two-week follow-up online survey in April 2021. An Internet survey company, Macromill, Inc. [28], recruited participants based on the following inclusion criteria: (a) those who live in Japan and whose native language is Japanese, (b) those aged ≥ 20 years, and (c) those who perceive themselves to be obese. The perception of obesity was assessed by the same question as in the original WBIS (Appendix for details). Macromill could access to over 2,000,000 monitors representing all prefecture in Japan. Participants of this study were drawn from their monitor registered as respondents of the company. Of the available respondents, 285 participants completed a web-based questionnaire in the baseline survey. After two weeks, the company invited the responders of the baseline survey to participate in the follow-up survey in order of arrival and 100 participants completed the follow-up survey. Responses were anonymous.

### Measures

Baseline survey included the Japanese version of the WBIS and scales for obesity-related quality of life, self-esteem, self-efficacy, and depression. Follow-up survey included the Japanese version of the WBIS.

The original version of the WBIS is an 11-item questionnaire that measures weight-related self-stigma and has a one-factor structure [15]. These items are scored on a 7-point Likert scale, ranging from 1 (strongly disagree) to 7 (strongly agree). Higher scores indicate more internalization of weight bias. The Japanese version of the WBIS was developed through three steps. First, two authors who were native Japanese speakers independently forward translated the original WBIS into Japanese and combined the two Japanese translations into one. Second, back-translation was conducted by a native English translator who was blinded to the original scale. Third, the original scale developer reviewed the English translation produced in the second step. Based on the suggestions of the developer, several items were modified by repeating the forward and backward translation procedures to reflect the original meaning after translation. Finally, we obtained the permission of the scale developer and used the Japanese version of the WBIS. The back-translation of the Japanese WBIS is provided in Appendix.

Obesity-related quality of life was evaluated using the Japanese version of the Obesity and Weight Loss Quality of Life Questionnaire (OWLQOL) [29, 30]. As the same as the original, it is composed of 17 items rated on a 5-point Likert scale. As a negative correlation was previously demonstrated between weight bias internalization and weight-related quality of life [31], a negative correlation would be expected between the WBIS and the OWLQOL.

Self-esteem was evaluated using the Japanese version [32] of the Rosenberg Self-Esteem Scale (RSES) [33]. As with the original, the Japanese version [32] is composed of 10 items rated on a 4-point Likert scale. A strong negative correlation was reported between the WBIS and the RSES [15].

Self-efficacy was evaluated using the General Self-Efficacy Scale (GSES) [34], which has 16 items rated on dichotomous (yes/no) scale. Weight bias internalization was reported to be associated with lower self-efficacy [35]; therefore, the present study predicted a negative correlation between the WBIS and the GSES.

Depression was evaluated using the Japanese version [36] of the Center for Epidemiologic Studies Depression Scale(CES-D) [37]. Both the original and its Japanese version [36] are composed of 20 items rated on a 4-point Likert scale. A strong positive correlation was reported between the WBIS and the CES-D [19].

### Statistical analyses

We calculated the Cronbach’s alpha for internal consistency and the intra-class correlation coefficient (ICC) for test-retest reliability. Confirmatory factor analysis (CFA) was also conducted for structural validity. In CFA, we assumed a one-factor model as observed in the previous study [38]. The indicators of model fit were chi-square, comparative fit index (CFI), root mean square error of approximation (RMSEA), and standardized root mean square residual (SRMR).　A good　fit was indicated by a value of 0.95 or more for the CFI, 0.06 or less for the RMSEA, and 0.08 or less for SRMR [39]. To evaluate convergent validity, we calculated Pearson’s correlation coefficients between the WBIS and OWLQOL, RSES, GSES, or CES-D in the baseline survey. The minimum effect size for detection in this study was 0.20. Based on a sample size calculation using G-power version 3.1.9.7, the necessary sample size was estimated to be more than 255 in the case of an alpha error probability of 0.05 and p power (1-β) of 0.90. In the test-retest reliability analysis, the sample size could be considered excellent when more than 100 participants were recruited [40]. Therefore, the numbers of participants in this study, 285 at baseline survey and 100 at follow-up survey, were adequate.

P-value below 0.05 was regarded as statistically significant. Data were analyzed using SPSS ver. 23, and CFA was performed using AMOS version 23.0.

## Results

Demographic characteristics of the participants at baseline and follow-up survey are in Table 1. In the baseline survey (N = 285, 151 females and 134 males, mean age = 47.7 ± 13.5), the mean BMI was 25.0 (SDs = 3.5). In BMI categories, about half of the participants were normal weight. Characteristics of the participants at the follow-up survey (N = 100, 55 females and 45 males, mean age = 46.5 ± 13.5) did not differ from the baseline survey.

**Table 1:** Demographic characteristics of the participants.

n (%)	Baseline survey N = 285	Follow-up survey N = 100
Gender		
Female	151 (53.0)	55 (55)
Male	134 (47.0)	45 (45)
Age category		
< 30	28 (9.8)	10 (10)
30–39	52 (18.2)	20 (20)
40–49	78 (27.4)	31 (31)
50–59	71 (24.9)	20 (20)
≥ 60	56 (19.6)	19 (19)
BMI category		
Underweight (< 18.50 kg/m2)	3 (1.1)	1 (1)
Normal-weight (18.50-24.99 kg/m2)	157 (55.1)	50 (50)
Overweight (25.00-29.99 kg/m2)	102 (35.8)	41 (41)
Obesity (> 30 kg/m2)	23 (8.1)	8 (8)
Education status		
Elementary/junior high school	9 (3.2)	3 (3)
High school	82 (28.8)	29 (29)
College	73 (25.6)	22 (22)
University	112 (39.3)	43 (43)
Graduate school	9 (3.2)	3 (3)
Marital status		
Married	178 (62.5)	64 (64)
Not married	107 (37.5)	36 (36)
Employment status		
Full-time worker	143 (50.2)	49 (49)
Part-time worker	26 (9.1)	7 (7)
Unemployed	69 (24.2)	25 (25)
Others	47 (16.5)	19 (19)

Cronbach’s alpha of the Japanese WBIS, as a measure of internal consistency, was 0.91 at baseline and 0.92 at follow-up. The ICC, as a measure of test-retest reliability, was 0.87 (p < 0.01) calculated using longitudinal data of 100 participants.

**Fig. 1:**
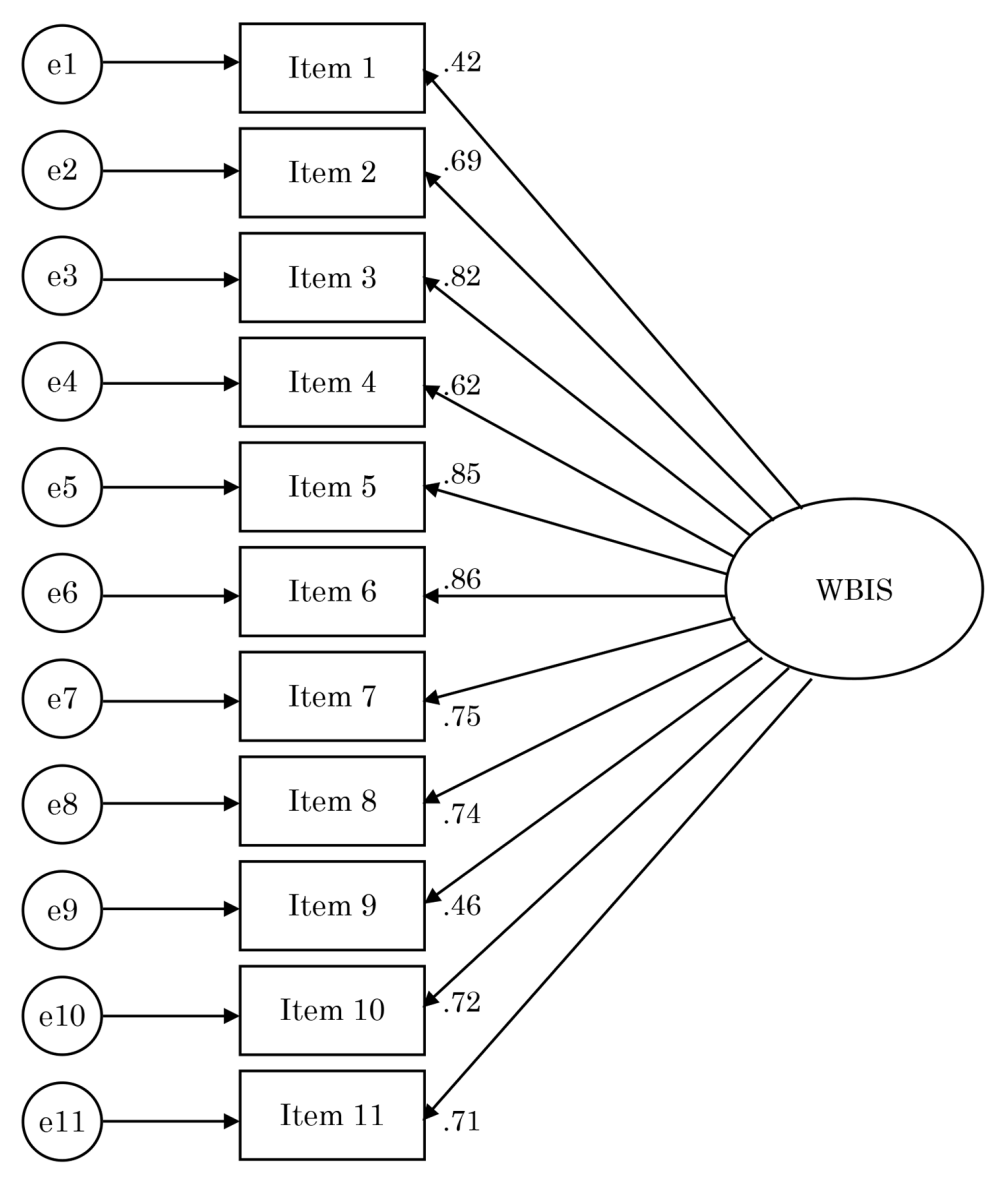
CFA path diagram of the Japanese version of the WBIS (N = 285). Factor loadings were standardized. χ2 (44) = 158.6, CFI = 0.93, RMSEA = 0.096, SRMR = 0.051

The results of CFA are shown in Fig. 1. The one-factor hypothesized model demonstrated acceptable fit (χ2 (44) = 158.6, CFI = 0.93, RMSEA = 0.096, SRMR = 0.051). Table 2 shows the correlation coefficients between the Japanese WBIS and OWLQOL, RSES, GSES, or CES-D. The total scores of the Japanese WBIS had a moderate positive correlation with CES-D, a moderate negative correlation with GSES, and strong negative correlations with OWLQOL and RSES.

**Table 2:** Convergent validity of the Japanese version of the WBIS (N = 285).

Variables	Mean (SD)	Correlation coefficient ^a^
WBIS	43.1 (12.1)	
OWLQOL	65.7 (18.9)	-0.84^※^
RSES	25.0 (5.6)	-0.66^※^
GSES	7.5 (4.7)	-0.45^※^
CES-D	14.7 (10.5)	0.48^※^

WBIS: Weight stigma internalization scale

OWLQOL: Obesity and Weight Loss Quality of Life Questionnaire

RSES: Rosenberg Self-Esteem Scale

GSES: General Self-Efficacy Scale

CES-D: Center for Epidemiologic Studies Depression Scale

^a^ Calculating Pearson's correlation coefficient with the Japanese version of the WBIS

^※^*P-*value < 0.05

## Discussion

The purpose of this study was to develop a Japanese version of the WBIS and to verify its reliability and validity. We finished the translation process and conducted the analysis. The results supported most of our hypotheses indicating that the Japanese WBIS showed good internal consistency, test-retest reliability, and convergent validity. The CFA showed acceptable results although RMSEA was higher than the cut off value of 0.06. This may be due to the sample size of the present study as RMSEA tends to be high with small sample size such as 250 [39].

The mean (SD) of the Japanese version WBIS was 43.1 (12.1), close to that of the original version [15]. The existence of obesity stigma was widely confirmed in Japan [27], although Japan has a low obesity rate. Social discrimination against people with obesity can lead to further obesity [40] and can prevent weight loss [41]. Flint et al., recommended that healthcare professionals working with people with obesity need to be educated about reducing weight bias internalization [42]. Cognitive-behavioral intervention study targeting weight stigma was conducted and produced short-term reductions in WBIS [43]. Measuring WBI is therefore needed both in society and in individuals to help reduce the number of people with obesity and overweight in Japan. The use of the Japanese version of the WBIS would be valuable to assess the levels of weight bias internalization in individuals in daily clinical practice and intervention studies.

In conclusion, we developed the Japanese version of the WBIS, and demonstrated that it has adequate psychometric properties, internal consistency, test-retest reliability, structural validity, and convergent validity for Japanese adults.

### Limitations

First, this study was conducted thorough an Internet-based survey, which might lead to a selection bias as those with high internalized weight stigma may be reluctant to participate in the Internet survey. In addition, the generalizability of this study could be questioned. Second, the response rate and dropout rate could not be calculated since the Internet survey company recruited participants until the target number of participants.

### Electronic supplementary material

Below is the link to the electronic supplementary material.

Supplementary Material 1

## Data Availability

The raw data generated during this study are included in Additional file 1. The datasets analyzed during the current study are available from the corresponding author on reasonable request.

## Appendix: The back-translation version of the Japanese WBIS

Question for screening survey participants.

How do you regard your own body weight?

1 = very underweight

2 = underweight

3 = slightly underweight

4 = average

5 = slightly overweight

6 = overweight

7 = very overweight

Only those participants who selected choices 5 through 7 were presented with the WBIS.

Questionnaire

How do you feel about the following statements? Please select the appropriate response1 = strongly disagree

2 = disagree

3 = slightly disagree

4 = neither agree nor disagree

5 = slightly agree

6 = agree

7 = strongly agree

1. As someone who is overweight, I think that I am as capable as other people
2. As a result of my weight, I am less attractive than most people
3. I am very conscious of what other people think about me, so I feel anxious about being overweight
4. I wish I could dramatically change my weight
5. I get depressed when I think about being overweight
6. I am fed up with being overweight
7. My weight is an important factor in defining my value as a person
8. So long as I am overweight, I think that I don’t deserve to lead a truly fulfilling social life
9. I think it is fine to stay at my current weight
10. Due to being overweight, I don’t feel that I am my true self
11. Due to my weight, I don’t know how someone attractive could be interested in me

## Abbreviations

WBIS: Weight stigma internalization scale.
OWLQOL: Obesity and weight loss quality of life questionnaire.
GSES: General Self-Efficacy Scale.
CES-D: Center for Epidemiologic Studies Depression Scale.
ICC: intra-class correlation coefficient.
CFA: confirmatory factor analysis.
CFI: comparative fit index.
RMSEA: root mean square error of approximation.
SRMR: standardized root mean square residual.
